# Enhanced Photoelectrochemical Performance of BiVO_4_ Photoanodes Co-Modified with Borate and NiFeO_x_

**DOI:** 10.3390/mi16080866

**Published:** 2025-07-27

**Authors:** Siqiang Cheng, Yun Cheng, Taoyun Zhou, Shilin Li, Dong Xie, Xinyu Li

**Affiliations:** 1 School of Information, Hunan University of Humanities, Science and Technology, Loudi 417000, Chinaxie.dong@139.com (D.X.); 2 Key Laboratory of Low-Dimensional Structural Physics and Application, Education Department of Guangxi Zhuang Autonomous Region, College of Physics and Electronic Information Engineering, Guilin University of Technology, Guilin 541004, China; lixinyu5260@163.com

**Keywords:** photoelectrochemical water splitting, BiVO_4_ photoanode, successive ionic layer adsorption and reaction (SILAR), borate surface modification, NiFeO_x_ cocatalyst

## Abstract

Despite significant progress in photoelectrochemical (PEC) water splitting, high fabrication costs and limited efficiency of photoanodes hinder practical applications. Bismuth vanadate (BiVO_4_), with its low cost, non-toxicity, and suitable band structure, is a promising photoanode material but suffers from poor charge transport, sluggish surface kinetics, and photocorrosion. In this study, porous monoclinic BiVO_4_ films are fabricated via a simplified successive ionic layer adsorption and reaction (SILAR) method, followed by borate treatment and PEC deposition of NiFeO_x_. The resulting B/BiVO_4_/NiFeO_x_ photoanode exhibits a significantly enhanced photocurrent density of 2.45 mA cm^−2^ at 1.23 V vs. RHE—5.3 times higher than pristine BiVO_4_. It also achieves an ABPE of 0.77% and a charge transfer efficiency of 79.5%. These results demonstrate that dual surface modification via borate and NiFeO_x_ is a cost-effective strategy to improve BiVO_4_-based PEC water splitting performance. This work provides a promising pathway for the scalable development of efficient and economically viable photoanodes for solar hydrogen production.

## 1. Introduction

The increasing depletion of fossil fuels and the growing environmental concerns have driven an urgent demand for clean and sustainable energy alternatives [1]. Among various solar-to-chemical conversion strategies, PEC systems have emerged as promising platforms for utilizing solar energy to drive oxidation and reduction reactions within a single device [2]. In particular, the PEC oxygen evolution reaction (OER) at the photoanode plays a crucial role in enabling water oxidation and other oxidative processes, and its efficiency significantly influences the overall system performance.

BiVO_4_ has been identified as one of the most promising photoanode materials, owing to its suitable bandgap (~2.4 eV), strong visible-light absorption, favorable valence band alignment for water oxidation, and relatively low cost [3,4]. Moreover, BiVO_4_ is composed of earth-abundant and environmentally benign elements, making it attractive for practical PEC applications. However, its PEC performance is still far below the theoretical limit due to inherent limitations such as poor charge carrier mobility, short carrier lifetimes (~40 ns), and high bulk and surface recombination rates [5,6,7,8].

To address these issues, a variety of strategies have been developed, including morphology engineering, heterojunction construction, elemental doping, surface cocatalyst loading, and plasmonic enhancement [9,10]. Among these, morphology engineering has proven particularly effective in reducing charge recombination by shortening the diffusion distance of photogenerated holes, which in BiVO_4_ is typically limited to ~70 nm [11]. Nanostructuring also improves the electrode–electrolyte interfacial area and facilitates more efficient charge separation and collection compared to dense films.

Various fabrication techniques have been explored to control the morphology of BiVO_4_ photoanodes, such as metal–organic decomposition [12], spray pyrolysis [13], spin coating [14], reactive sputtering [15], dip coating [16], electrodeposition [17], and chemical bath deposition via SILAR method [18]. Among them, the SILAR method, first introduced by Nicolau in 1984, has gained particular attention due to its low cost, operational simplicity, and capability to produce conformal and uniform thin films under ambient conditions.

For instance, Guo et al. [19] reported the preparation of porous BiVO_4_ thin films via a simplified SILAR approach, which yielded a photocurrent density of 0.70 mA·cm^−2^ at 1.23 V vs. RHE. However, even with further surface modifications, the photocurrent remained below 1.0 mA·cm^−2^, indicating that both bulk and interfacial recombination continued to restrict performance.

To further mitigate surface charge losses, post-synthetic surface modification strategies have been developed. Among them, borate treatment has proven effective in passivating surface trap states. Meng et al. [20] demonstrated that the in situ formation of [B(OH)_4_]^−^ species on the BiVO_4_ surface can significantly reduce surface recombination and enhance charge injection, resulting in improved PEC performance. Meanwhile, the integration of transition metal-based cocatalysts, such as NiFeO_x_, has also been widely investigated. NiFeO_x_ exhibits high catalytic activity toward the OER, where Ni functions as the redox-active center forming NiOOH intermediates, while Fe promotes charge transfer and facilitates intermediate adsorption, together accelerating surface reaction kinetics.

Despite these individual advances, studies combining both borate passivation and cocatalyst modification in a single system remain limited, particularly with an emphasis on low-cost and scalable synthesis routes. Furthermore, few reports provide systematic comparisons of the contributions from each modification step to overall PEC performance.

In this work, we report a cost-effective and scalable strategy for enhancing the PEC performance of BiVO_4_ photoanodes through co-modification with borate and NiFeO_x_. BiVO_4_ thin films were first synthesized using a simplified SILAR approach, followed by borate surface treatment and photo-assisted electrodeposition of NiFeO_x_ as a cocatalyst. The resulting B/BiVO_4_/NiFeO_x_ photoanode exhibited a photocurrent density of 2.45 mA·cm^−2^ at 1.23 V vs. RHE and an onset potential as low as 0.31 V. Additionally, it maintained 79.5% of its initial photocurrent after 5 h of continuous illumination and achieved an applied bias photon-to-current efficiency (ABPE) of 0.78%.

These enhancements are attributed to the synergistic effects of borate-induced surface passivation and NiFeO_x_-mediated catalytic promotion, which together suppress charge recombination and facilitate efficient hole extraction and interfacial oxygen evolution. This work provides valuable insights into low-cost interface engineering and offers a practical pathway toward the scalable development of high-performance photoanodes for PEC applications.

## 2. Materials and Methods

### 2.1. Materials

All chemical reagents used in this study were of analytical grade and employed without further purification. Bismuth nitrate pentahydrate (Bi(NO_3_)_3_·5H_2_O) was purchased from Aladdin. Ferrous sulfate (FeSO_4_) was supplied by Ron, and deionized water (H_2_O) was obtained from Wahaha. Other reagents, including potassium iodide (KI), vanadyl acetylacetonate (VO(acac)_2_), p-benzoquinone (C_6_H_4_O_2_), nickel sulfate hexahydrate (NiSO_4_·6H_2_O), dimethyl sulfoxide (DMSO, C_2_H_6_OS), ammonium metavanadate (NH_4_VO_3_), anhydrous sodium sulfite (Na_2_SO_3_), nitric acid (HNO_3_), anhydrous ethanol (C_2_H_5_OH), sodium hydroxide (NaOH), and hydrogen peroxide (H_2_O_2_), were all commercially available and used as received.

All chemicals involved in the synthesis—such as boric acid, nickel nitrate, and iron nitrate—are inexpensive, abundant, and commonly available. The fabrication process requires only simple aqueous-phase treatments and low-temperature annealing, without the need for vacuum equipment, inert atmospheres, or precious metals.

Based on laboratory-scale estimates, the total material cost for preparing a 1 cm^2^ B/BiVO_4_/NiFeO_x_ photoanode is approximately $0.02–0.05. This is significantly lower than that of noble-metal-based cocatalyst systems (e.g., Pt or IrO_2_), which typically exceed $0.50 per cm^2^ and involve complex deposition techniques such as sputtering or atomic layer deposition. Therefore, the proposed approach offers a cost-effective and scalable pathway for developing efficient photoanodes suitable for practical PEC water-splitting applications.

### 2.2. Experimental Instruments

A range of advanced analytical and characterization instruments was employed to ensure the accuracy and reliability of material synthesis, structural analysis, and PEC performance evaluation throughout the study. The major experimental equipment, including their model numbers and manufacturers, is summarized in Table 1.

### 2.3. Preparation of BiVO_4_ Electrodes

Fluorine-doped tin oxide (FTO) glass substrates were ultrasonically cleaned in deionized water and ethanol for 10 min each, followed by gentle wiping with ethanol-soaked tissues. The electrodeposition electrolyte was prepared by dissolving 0.4 M potassium iodide (KI) in 50 mL of deionized water, adjusting the pH to 1.7 with nitric acid, and subsequently adding 0.4 M bismuth nitrate pentahydrate [Bi(NO_3_)_3_·5H_2_O]. The solution was magnetically stirred until a uniform orange-red solution was obtained. Separately, 0.2 M p-benzoquinone was dissolved in 20 mL of anhydrous ethanol and sonicated for 5 min to ensure full dispersion. The two solutions were then mixed and stirred for an additional 5 min to form a homogeneous electrolyte.

Electrodeposition was conducted in a standard three-electrode system with FTO as the working electrode, a platinum sheet as the counter electrode, and a saturated Ag/AgCl electrode as the reference. The mixed electrolyte containing KI, Bi(NO_3_)_3_·5H_2_O, and p-benzoquinone was used, and electrodeposition was carried out at −0.11 V vs. Ag/AgCl for 5 min, forming a BiOI precursor layer on the FTO surface.

To obtain BiVO_4_, 50 μL of 0.2 M vanadyl acetylacetonate [VO(acac)_2_] in dimethyl sulfoxide (DMSO) was drop-cast onto the BiOI layer, followed by annealing at 450 °C for 2 h in a muffle furnace. The resulting film was immersed in 1 M NaOH to remove excess V_2_O_5_, rinsed with deionized water, and air-dried.

### 2.4. Preparation of B/BiVO_4_/NiFeO_x_ Photoelectrodes

**Figure 1:**
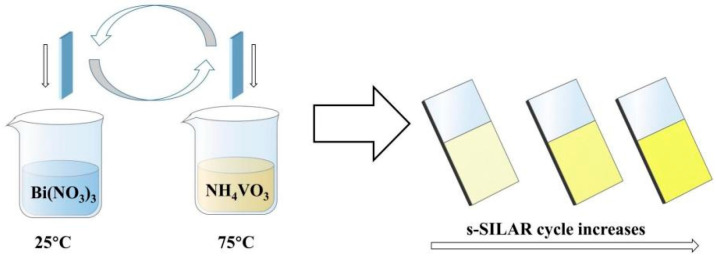
The schematic illustration of the fabrication of the B/BiVO_4_/NiFeO_x_ electrode.

Monoclinic BiVO_4_ photoanodes were synthesized using a simplified successive ionic layer adsorption and reaction (s-SILAR) method, modified from Guo et al. [19]. As shown in Figure 1, two precursor solutions were prepared: (1) 20 mL of 25 mM Bi(NO_3_)_3_·5H_2_O in water, adjusted with glacial acetic acid (volume ratio 1:19), and (2) 20 mL of 25 mM NH_4_VO_3_ solution, maintained at 75 °C under constant stirring.

Clean FTO substrates were sequentially immersed in the Bi^3+^ solution for 20 s and the V^5+^ solution for 20 s. After each cycle, the substrates were briefly exposed to air to facilitate reaction via residual thermal energy. One immersion sequence constituted one s-SILAR cycle. The process was repeated for 10, 20, and 30 cycles to obtain BiVO_4_ films of increasing thickness, denoted as BiVO_4_-10, BiVO_4_-20, and BiVO_4_-30, respectively. After deposition, the samples were rinsed with deionized water, air-dried, and annealed at 450 °C for 2 h (heating rate: 10 °C/min). Residual impurities were removed by immersion in 1 M NaOH.

B-doped BiVO_4_ (B/BiVO_4_) films were obtained by immersing the BiVO_4_ electrodes in 0.5 M boric acid solution (pH 9.5, adjusted with KOH) for 12 h, followed by rinsing with deionized water.

NiFeO_x_ cocatalyst was deposited via photo-assisted electrodeposition. A mixed solution of 0.4 M FeSO_4_ and 0.04 M NiSO_4_ was prepared, and deposition was carried out under light illumination at 0.6 V vs. Ag/AgCl for 180 s in a three-electrode system. The resulting B/BiVO_4_/NiFeO_x_ electrodes were rinsed and air-dried. For comparison, BiVO_4_/NiFeO_x_ electrodes were prepared under identical conditions without B doping.

### 2.5. PEC Characterization and Evaluation Methods of Photoanodes

PEC measurements were performed using a CHI 760E electrochemical workstation (CHI 760E, CH Instruments, Shanghai, China). Simulated AM 1.5G sunlight (100 mW·cm^−2^) was provided by a xenon lamp (PLS-SXE300+, Beijing Perfectlight Technology Co., Ltd., Beijing, China) directed onto the back side of the photoanodes. A standard three-electrode configuration was used, comprising BiVO_4_-based photoanodes as the working electrode, a platinum foil as the counter electrode, and a saturated calomel electrode (SCE) as the reference. Electrolytes included 0.5 M Na_2_SO_4_ (pH 7) and 0.1 M H_3_BO_3_ (pH 9.5), with or without 0.5 M Na_2_SO_3_ as a hole scavenger.

Linear sweep voltammetry (LSV) was conducted from −0.5 to 0.7 V vs. SCE at 10 mV·s^−1^. Electrochemical impedance spectroscopy (EIS) was carried out at 0.75 V vs. RHE with a 10 mV AC perturbation over the 100 kHz to 0.1 Hz frequency range. Transient photocurrent and stability tests were recorded under chopped illumination using the *i*–*t* method.

All measured potentials are converted to the reversible hydrogen electrode (RHE) scale using Equation (1):

(1) E(vs⋅RHE)=E(vs⋅SCE)+0.241+0.0591×PH

where, E(vs⋅SCE) is the recorded potential vs. SCE, and 0.241 is the standard potential of SCE at room temperature.

The surface charge transfer efficiency ($\eta_{transfer}$) is an important indicator of charge separation at the semiconductor/electrolyte interface, which can be calculated as:

(2) $$\eta_{transfer}=\frac{J_{H_{2}O}}{J_{abs}\times \eta_{bulk}}\times 100\%$$

where, $J_{abs}$ is theoretical maximum current density from light absorption, $J_{H_{2}O}$ is actual photocurrent density generated by photoelectrode in the electrolyte (mA·cm^−2^), $\eta_{bulk}$ is the charge separation rate yield of the semiconductor.

Assuming a Faradaic efficiency of 100%, the applied bias photon-to-current efficiency (ABPE) is calculated as follows:

(3) $$ABPE=\frac{J_{H_{2}O}\times (1.23-V_{app})}{plight}\times 100\%$$

where, $V_{app}$ is the applied potential vs. RHE, and plight is the light power density (100 mW·cm^−2^).

## 3. Results and Analysis

### 3.1. Characterization of BiVO_4_ Photoanode

**Figure 2:**
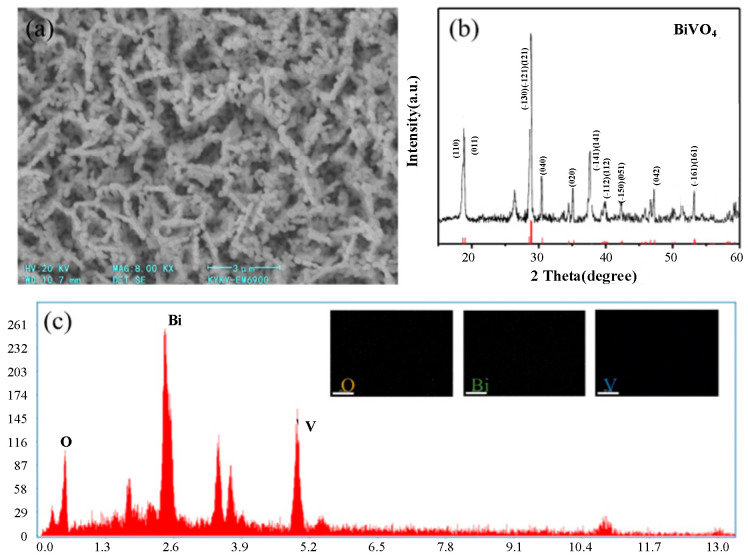
BiVO_4_ photoanode (**a**) SEM, (**b**) XRD analysis, and (**c**) EDS analysis, inset: elemental distribution of O, Bi, and V.

Characterization of BiVO_4_ Photoanode was shown in Figure 2. SEM analysis was carried out, as shown in Figure 2a, the BiVO_4_ film consists of a large number of irregularly shaped nanoparticles that are densely packed and stacked to form a porous architecture. This porous morphology was attributed to the transformation of the precursor BiOI.

To further confirm the crystal structure of the synthesized BiVO_4_, XRD analysis was carried out. As shown in Figure 2b, the main diffraction peaks located at 18.91° (011), 28.80° (112), 30.60° (004), 34.50° (200), and 47.10° (024) are well indexed to the monoclinic scheelite phase of BiVO_4_ (JCPDS No. 14-0688), indicating successful phase formation. No additional peaks corresponding to secondary phases of bismuth or vanadium oxides are detected, confirming the high crystallinity and phase purity of the BiVO_4_ film. It is worth noting that a peak observed near 26.7° does not originate from BiVO_4_ but is attributed to the (110) reflection of the FTO substrate, which is commonly used as the conductive support in photoanode fabrication. Elemental composition and distribution are analyzed using energy-dispersive X-ray spectroscopy (EDS), as shown in Figure 2c; the elements are uniformly distributed throughout the surface, indicating homogeneous composition.

**Figure 3:**
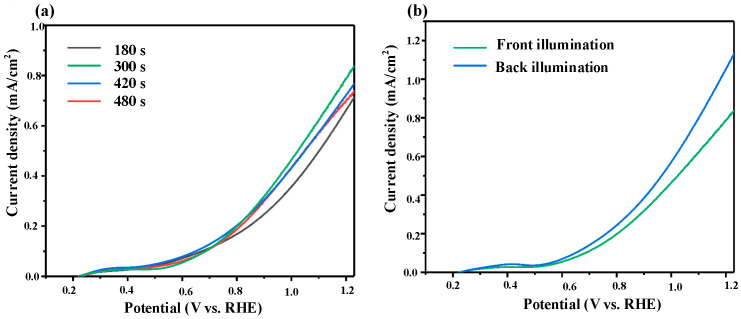
(**a**) LSV curves of BiVO_4_ thin films prepared with different electrodeposition times, and (**b**) LSV curves for frontal and backside illumination.

To evaluate the impact of deposition time on PEC performance, LSV was conducted under AM 1.5G illumination. As shown in Figure 3a, the photocurrent densities at 1.23 V vs. RHE for films deposited for 180 s, 300 s, 420 s, and 480 s are 0.71, 0.84, 0.77, and 0.74 mA·cm^−2^, respectively. These results indicate an initial enhancement in PEC performance followed by a slight decline as the deposition time increases. The film deposited for 300 s exhibits the highest PEC activity, balancing sufficient light absorption with efficient charge separation and transport.

To further verify this hypothesis, front-side and back-side illumination tests were performed (Figure 3b). At 1.23 V vs. RHE, the photocurrent densities under front and back illumination are 0.84 and 1.13 mA·cm^−2^, respectively. The higher photocurrent observed under back-side illumination confirms that charge carriers generated closer to the conductive substrate have a greater probability of being collected before recombination. Based on these findings, all subsequent two-step fabrication procedures for BiVO_4_ photoanodes are standardized using a 300 s electrodeposition and back-side illumination setup.

### 3.2. Characterization of B/BiVO_4_/NiFeO_x_; Photoelectrodes

#### 3.2.1. Morphological Structure Analysis

**Figure 4:**
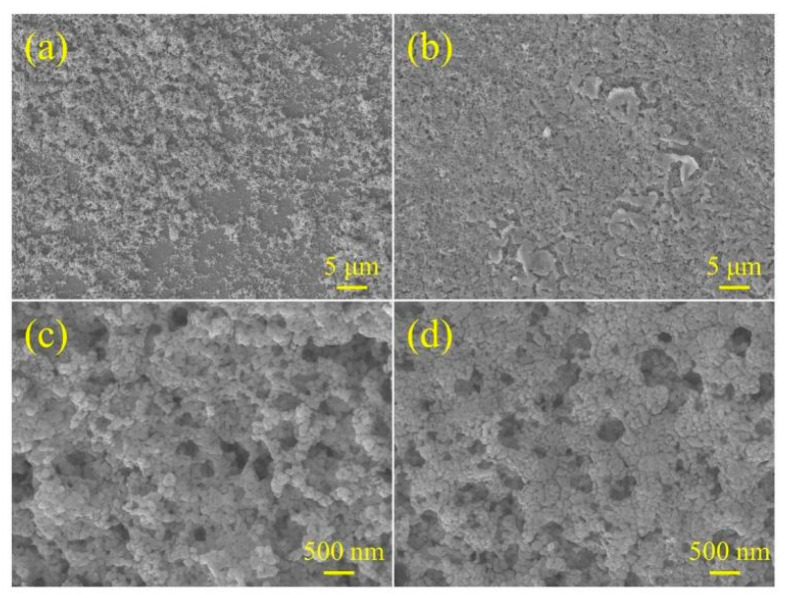
(**a**) SEM of BiVO_4_ prepared by 10 cycles of s-SILAR, (**b**) SEM of BiVO_4_ prepared by 20 cycles of s-SILAR, (**c**) high-magnification (scale bar: 500 nm) of BiVO_4,_ and (**d**) high-magnification (scale bar: 500 nm) of B/BiVO_4_/NiFeO_x_.

Figure 4 presents the surface morphology evolution of BiVO_4_ films fabricated with different numbers of s-SILAR cycles, as well as the corresponding composite films, observed via SEM.

Figure 4a exhibits a porous structure composed of agglomerated particles of varying sizes. This morphology is primarily attributed to the formation of a BiVO_4_ “seed layer” on the FTO substrate during the initial deposition cycles. Subsequent deposition preferentially occurs on these seeds, leading to a layered particle accumulation. Due to the relatively low number of deposition cycles, the overall film thickness is limited, and partial exposure of the FTO substrate can be observed, indicating insufficient surface coverage. In Figure 4b, a noticeable change in morphology is observed: the particles are more densely packed, and the film structure becomes more compact while still retaining a certain degree of porosity. The enhanced compactness suggests that increasing the number of deposition cycles facilitates the formation of a more continuous and uniform film, thereby improving coverage and structural stability. Figure 4c and Figure 4d provide high-magnification (scale bar: 500 nm) images of the samples in Figure 4a and Figure 4b, respectively. From a microscopic perspective, the particle morphology and porous structure in Figure 4c,d remain largely unchanged, and no significant morphological differences are observed. This indicates that the loading of B and NiFeO_x_ is relatively low and does not notably alter the overall surface morphology.

To evaluate the surface morphology and elemental distribution of the B/BiVO_4_/NiFeO_x_ composite, SEM and EDS analysis were conducted. The SEM observations confirmed a relatively uniform and compact film morphology, while the EDS analysis revealed the successful incorporation of boron (B), nickel (Ni), and iron (Fe) into the BiVO_4_ matrix. As shown in Table 2, the atomic percentage of B reaches 57.60%, with a corresponding weight percentage of 24.12%, indicating a substantial presence of borate species on the surface. This may be attributed to the strong adsorption affinity of borate ions and their tendency to form surface-enriched layers during the modification process.

Although the atomic contents of Ni (1.87%) and Fe (2.70%) are comparatively low, their presence, confirmed by EDS, suggests that the NiFeO_x_ cocatalyst was effectively deposited onto the BiVO_4_ surface. Despite their low content, such cocatalysts are known to play a critical role in promoting surface reaction kinetics. The uniform distribution of B, Ni, and Fe across the film—verified by the EDS analysis—further confirms the successful co-modification of the BiVO_4_ photoanode.

These results collectively demonstrate that both borate passivation and NiFeO_x_ cocatalyst loading were effectively achieved, laying a solid foundation for the enhanced photoelectrochemical performance observed in subsequent measurements.

#### 3.2.2. Optical Property Analysis

**Figure 5:**
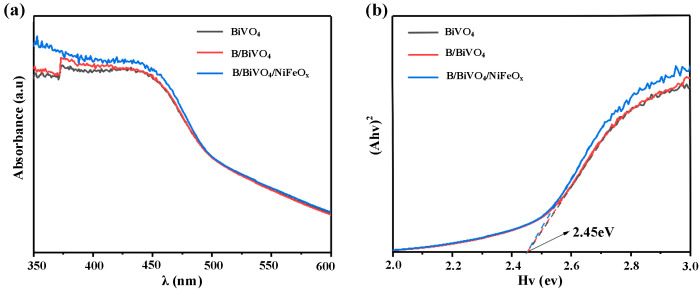
(**a**) UV-Vis absorption spectra of BiVO_4_, B/BiVO_4,_ and B/BiVO_4_/NiFeO_x_, (**b**) Tauc curve.

Figure 5 presents the UV–visible absorption spectra and the corresponding Tauc plots of BiVO_4_, B/BiVO_4_, and B/BiVO_4_/NiFeO_x_. As shown in Figure 5a, all samples exhibit strong absorption in the 350–500 nm wavelength range, with comparable absorption edge positions. This indicates that neither borate surface treatment nor NiFeO_x_ ocatalyst deposition significantly affects the intrinsic light-harvesting properties of BiVO_4_. The absence of a noticeable red-shift or blue-shift in the absorption edge further suggests that no new mid-gap states or structural transitions are introduced during the modification processes [21,22].

Tauc plots are constructed with Equation (4):

(4) $${\left(\alpha hv\right)}^{\frac{1}{n}}=A(hv-E_{g})$$

where α is the absorption coefficient, *hv* is the photon energy (*h* is a Planck’s constant and *v* is frequency), and *n* is a constant related to the nature of the band gap (with *n* = 1/2 for direct band gap semiconductors, which applies here).

As illustrated in Figure 5b, the estimated optical band gap of pristine BiVO_4_ is approximately 2.45 eV, which is consistent with previously reported values for monoclinic BiVO_4_ [11]. Notably, the band gap values for B/BiVO_4_ and B/BiVO_4_/NiFeO_x_ remain nearly identical to those of the pristine sample. This observation confirms that the applied modifications—borate passivation and NiFeO_x_ loading—occur primarily at the surface level and do not influence the bulk electronic structure or the optical transition energy of BiVO_4_. This is in agreement with previous studies, where surface engineering strategies were found to improve charge separation and interfacial kinetics without altering the fundamental band structure of the semiconductor [11].

These findings also support the conclusion that the enhanced PEC performance observed in the modified electrodes arises from improved interfacial properties—such as reduced recombination and enhanced surface reaction kinetics—rather than changes in the optical absorption or band gap energy.

#### 3.2.3. Crystal Structure Analysis

**Figure 6:**
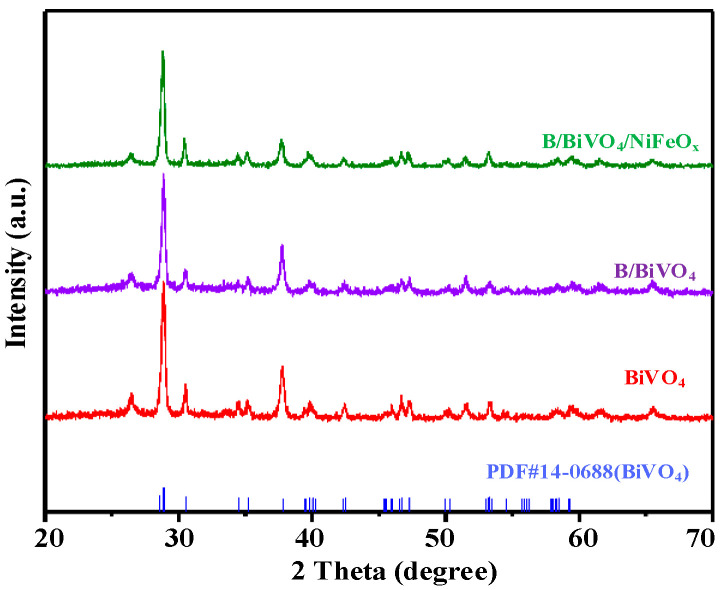
XRD spectra of BiVO_4_, B/BiVO_4_, and B/BiVO_4_/NiFeO_x_ photoanodes.

Figure 6 displays the XRD patterns of BiVO_4_, B/BiVO_4_, and B/BiVO_4_/NiFeO_x_ photoanodes. All samples exhibit diffraction peaks that are in good agreement with the standard pattern of monoclinic scheelite BiVO_4_ (PDF#14-0688). The main characteristic peaks are observed at 28.9° (121), 30.5° (040), 34.5° (200), 46.7° (240), and 53.3° (161), confirming the successful formation of the monoclinic BiVO_4_ phase with a well-defined and pure crystal structure. Notably, all three samples show highly similar XRD patterns, with no significant peak shifts or the emergence of new diffraction peaks. This suggests that B doping and NiFeO_x_ co-deposition do not induce a phase transformation or lead to lattice substitution. The absence of additional peaks in the XRD patterns suggests that the B, Ni, and Fe species introduced through surface modification either exist in amorphous form, have low crystallinity, or are presented as surface layers below the XRD detection threshold. The high atomic percentage of B observed in EDS likely reflects surface-enriched borate species rather than bulk incorporation [23,24].

#### 3.2.4. Elemental Composition Analysis

**Figure 7:**
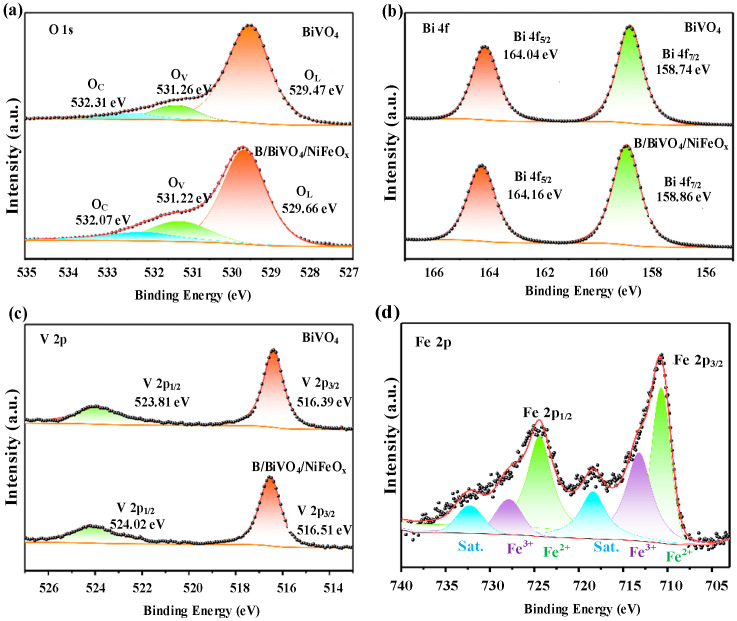
XPS of BiVO_4_ and B/BiVO_4_/NiFeO_x_: (**a**) O 1s, (**b**) Bi 4f, (**c**) V, (**d**) Fe.

To further investigate the chemical states and bonding environments of the elements in the photoanodes, high-resolution XPS analysis was conducted on both pristine BiVO_4_ and the B/BiVO_4_/NiFeO_x_ composite, as shown in Figure 7. As illustrated in Figure 7a, the O 1s spectra of both samples can be deconvoluted into three main components located at 529.47 eV (O_L_), 531.26 eV (O_V_), and 532.31 eV (O_C_), which correspond to lattice oxygen (O^2^^−^), oxygen vacancies or surface hydroxyl groups, and chemisorbed oxygen species (typically from dissociated or adsorbed water molecules), respectively [25]. Notably, the relative intensity of the O_V_ peak increases significantly in the B/BiVO_4_/NiFeO_x_ sample, indicating a higher concentration of oxygen vacancies induced by the surface modification process.

The formation of oxygen vacancies can be attributed to multiple factors. During the borate treatment and subsequent annealing, partial substitution or extraction of lattice oxygen atoms may occur, particularly at the Bi–O or V–O coordination sites, leading to the generation of oxygen-deficient regions. Additionally, the incorporation of NiFeO_x_ through photo-assisted electrodeposition can facilitate charge transfer at the interface, further promoting structural relaxation and vacancy formation. Similar mechanisms have been reported in the literature, where mild chemical or thermal treatments introduced oxygen vacancies that enhance charge transport and surface activity [26].

Functionally, oxygen vacancies play two important roles in improving PEC performance:

- Enhanced electronic conductivity—Oxygen vacancies introduce donor-like states near the conduction band, thereby improving charge carrier mobility and facilitating electron transport within the semiconductor.
- Improved surface reaction kinetics—Oxygen-deficient sites can act as active centers for water oxidation, promoting the adsorption and activation of OH^−^ intermediates and accelerating the OER.

In Figure 7b, two peaks at 164.04 eV (Bi 4f_5_/_2_) and 158.74 eV (Bi 4f_7_/_2_) are observed in pristine BiVO_4_, corresponding to Bi^3+^ [27]. In the B/BiVO_4_/NiFeO_x_ sample, these peaks shift slightly to 164.16 eV and 158.86 eV, respectively, indicating a more electron-deficient environment around Bi atoms, likely due to the electron-withdrawing effect of surface-deposited NiFeO_x_. In Figure 7c, pristine BiVO_4_ exhibits two distinct peaks at 523.81 eV (V 2p_1_/_2_) and 516.39 eV (V 2p_3_/_2_), corresponding to the V^5+^ oxidation state [28]. After NiFeO_x_ modification, these peaks shift slightly to 524.02 eV and 516.51 eV, respectively, suggesting electronic redistribution in the vanadium environment, possibly due to interfacial interactions with NiFeO_X_ and the presence of nearby oxygen vacancies. In Figure 7d, four peaks at 710.71 eV and 724.43 eV(assigned to Fe^2+^ 2p_3_/_2_ and 2p_1_/_2_), and 713.22 eV and 727.88 eV(assigned to Fe^3+^ 2p_3_/_2_ and 2p_1_/_2_), along with their corresponding satellite signals [29]. The coexistence of Fe^2+^ and Fe^3+^ species confirms the formation of a mixed-valence NiFeO_x_ cocatalyst.

It is worth noting that the Ni 2p XPS signal is extremely weak due to its low atomic concentration (~1.87%, as shown in Table 2). Moreover, the Ni 2p peaks are overlapped significantly with background noise and satellite structures, making reliable deconvolution and chemical state analysis challenging. Therefore, to maintain data clarity and accuracy, the Ni XPS spectrum is not included in Figure 7. Nonetheless, the successful co-deposition of Ni, as confirmed by EDS, together with the observed PEC enhancements, strongly supports the effective integration of NiFeO_x_ into the BiVO_4_ system.

### 3.3. Electrochemical Performance Analysis

All electrochemical measurements were carried out under simulated AM 1.5G sunlight in a standard three-electrode configuration using 0.1 M borate buffer solution (pH 9.5) as the electrolyte.

#### 3.3.1. PEC Performance

**Figure 8:**
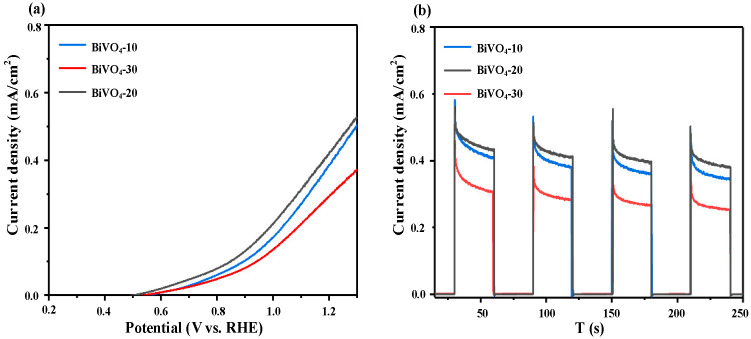
LSV curves (**a**) and transient photocurrents (**b**) of BiVO_4_ photoanodes prepared with different numbers of s-SILAR cycles.

Figure 8 presents the PEC performance of BiVO_4_ photoanodes fabricated with different deposition cycles using the s-SILAR method. As shown in Figure 8a, the photocurrent densities of BiVO_4_-10, BiVO_4_-20, and BiVO_4_-30 at 1.23 V vs. RHE are 0.42 mA·cm^−2^, 0.46 mA·cm^−2^, and 0.32 mA·cm^−2^, respectively. An increase in the number of deposition cycles from 10 to 20 significantly enhances the photocurrent, indicating improved light-harvesting ability with moderately increased film thickness. However, a further increase to 30 cycles leads to a decline in photocurrent, which can be attributed to excessive film thickness hindering charge separation and transport. All samples in Figure 8b exhibit sharp photocurrent spikes upon light-on/off switching, followed by a rapid decay, suggesting the presence of charge recombination at the electrode surface or interfaces. Among them, the BiVO_4_-20 sample shows the highest and most stable photocurrent response across multiple on/off cycles, consistent with the LSV results.

**Figure 9:**
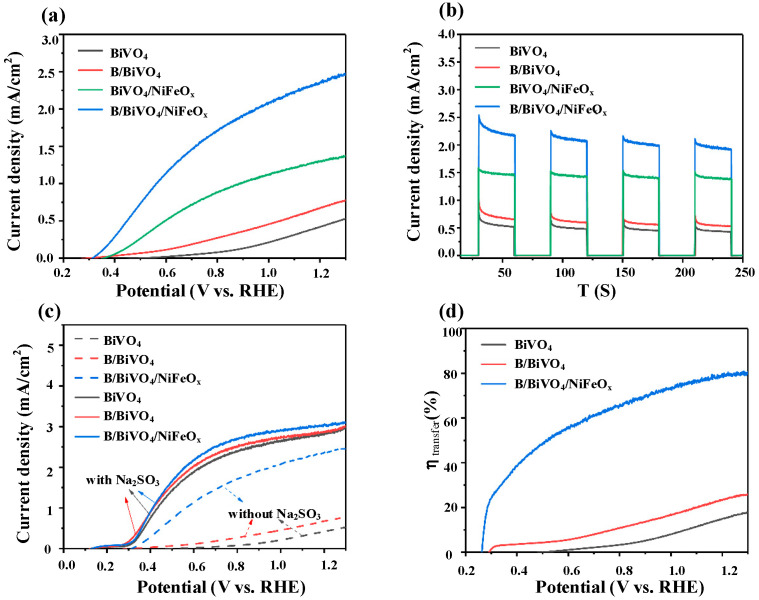
(**a**) LSV curves of BiVO_4_, B/BiVO_4_, BiVO_4_/NiFeO_x_, and B/BiVO_4_/NiFeO_x_, (**b**) transient photocurrent curves, (**c**) LSV curves of BiVO_4_, B/BiVO_4_, and B/BiVO_4_/NiFeO_x_ with vs. without Na_2_SO_3_, and (**d**) surface charge transfer efficiency.

Figure 9 compares the PEC performance of pristine BiVO_4_ and BiVO_4_ photoanodes modified with boron treatment (B) and NiFeO_x_ dual co-catalyst. As shown in Figure 9a, pristine BiVO_4_ exhibits an onset potential of 0.50 V vs. RHE and a photocurrent density of 0.46 mA·cm^−2^ at 1.23 V vs. RHE. B/BiVO_4_ increases the photocurrent to 0.70 mA·cm^−2^, while BiVO_4_/NiFeO_x_ enhances it to 1.32 mA·cm^−2^. B/BiVO_4_/NiFeO_x_ yields the best performance, with a positively shifted onset potential (0.31 V vs. RHE) and a maximum photocurrent of 2.45 mA·cm^−2^—representing 5.3×, 3.6×, and 1.9× improvements over pristine BiVO_4_, B/BiVO_4_, and BiVO_4_/NiFeO_x_, respectively.

This remarkable enhancement is attributed to the synergistic effect of boron-induced surface passivation and the electrocatalytic activity of NiFeO_x_. Boron treatment effectively reduces surface trap states and improves interfacial charge transfer, while NiFeO_x_ acts as a highly active oxygen evolution cocatalyst. In particular, Ni sites undergo redox cycling (Ni^2+^/Ni^3+^/Ni^4+^), forming NiOOH species that serve as the main OER active centers. The incorporation of Fe modulates the electronic structure of the Ni sites and facilitates the adsorption and conversion of oxygen intermediates (*OH, *O, *OOH), thereby accelerating surface reaction kinetics. Together, these effects promote efficient hole extraction from BiVO_4_, suppress surface recombination, and lower the overpotential for water oxidation [30].

Pristine BiVO_4_ in Figure 9b displays rapid decay upon illumination, indicating severe surface recombination. In contrast, BiVO_4_/NiFeO_x_ and B/BiVO_4_/NiFeO_x_ exhibit stronger and more stable photocurrents, confirming the beneficial role of NiFeO_x_ in enhancing charge separation and surface reaction kinetics. To further assess surface charge transfer, Figure 9c presents LSV curves with and without Na_2_SO_3_ as a hole scavenger. All samples exhibit increased photocurrent in the presence of Na_2_SO_3_, but B/BiVO_4_/NiFeO_x_ maintains a high current even without it, highlighting improved interfacial charge transfer. Figure 9d summarizes the surface charge transfer efficiency, calculated from photocurrent ratios at 1.23 V vs. RHE. Pristine BiVO_4_, B/BiVO_4_, and B/BiVO_4_/NiFeO_x_ show efficiencies of 15.9%, 24.3%, and 79.5%, respectively, clearly demonstrating the effectiveness of dual modification in enhancing surface charge transfer [31].

#### 3.3.2. Electrochemical Impedance Spectroscopy (EIS)

**Figure 10:**
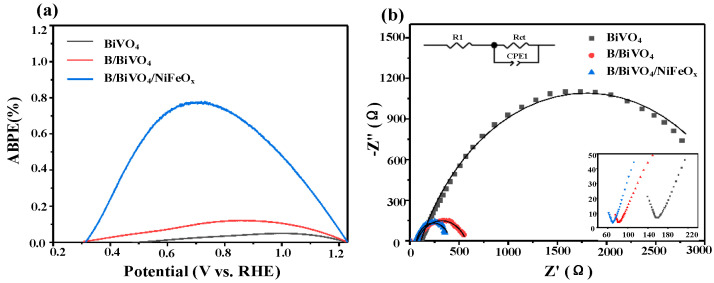
(**a**) ABPE, (**b**) impedance map. (Illustration) Amplified EIS in the low-frequency region.

Figure 10a presents the ABPE curves of differently modified BiVO_4_ photoanodes, calculated based on the previously obtained LSV data. The results indicate that the B/BiVO_4_/NiFeO_x_ photoanode exhibits a maximum ABPE of 0.77% at 0.66 V vs. RHE, which is significantly higher than those of B/BiVO_4_ (0.12% at 0.85 V) and pristine BiVO_4_ (0.05% at 1.00 V), corresponding to enhancements of approximately 6.4-fold and 15.4-fold, respectively.

To further investigate the interfacial charge transfer behavior, EIS is performed on the three photoanodes, with the resulting Nyquist plots shown in Figure 10b. The inset displays an enlarged view of the high-frequency region. The equivalent circuit model used for fitting includes a series resistance (R_s_), interfacial charge transfer resistance (R_ct), and a constant phase element (CPE). The two typical semicircles in the Nyquist plots correspond to charge transfer processes in the depletion layer of the semiconductor (high-frequency region) and at the semiconductor/electrolyte interface (low-frequency region), respectively [32,33,34]. Among the samples, B/BiVO_4_/NiFeO_x_ exhibits the smallest arc radius in the Nyquist plot, indicating the lowest interfacial charge transfer resistance, consistent with its superior photocurrent density and highest ABPE. In contrast, the pristine BiVO_4_ electrode shows the largest arc, suggesting limited charge transport capability and sluggish interfacial reaction kinetics. Although the B/BiVO_4_ sample exhibits reduced impedance compared to pristine BiVO_4_, its resistance remains higher than that of B/BiVO_4_/NiFeO_x_, further confirming the crucial role of NiFeO_x_ in facilitating charge transfer across the hotoanode/ electrolyte interface. EIS fitting data for each photoelectrode is shown in Table 3.

**Figure 11:**
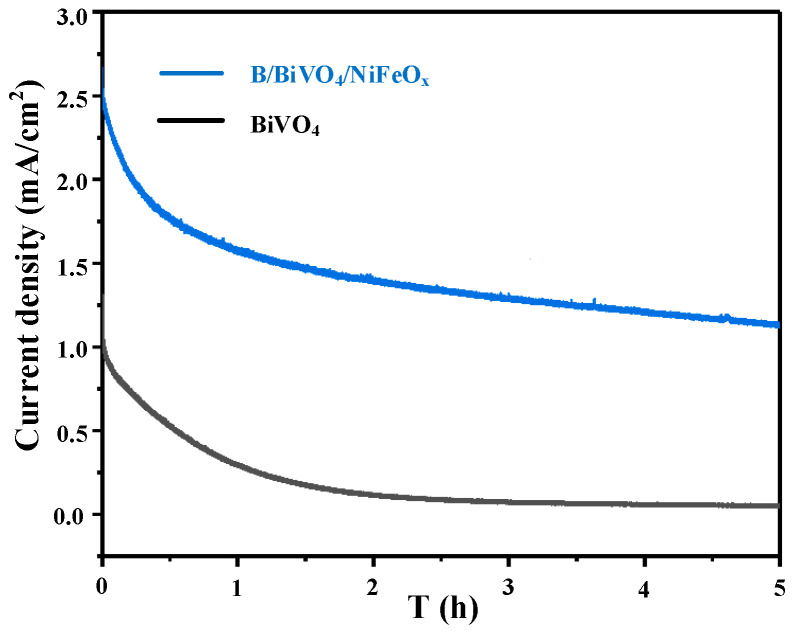
Stability Test.

Figure 11 presents the stability evaluation of the BiVO_4_ and B/BiVO_4_/NiFeO_x_ photoanodes under continuous illumination at a constant applied potential for 5 h. At the beginning of the measurement, the B/BiVO_4_/NiFeO_x_ photoanode exhibits a significantly higher photocurrent density of approximately 2.5 mA cm^−2^, compared to 1.4 mA cm^−2^ for pristine BiVO_4_. As the testing time progresses, both electrodes show a gradual decline in photocurrent density. After 5 h of continuous operation, the B/BiVO_4_/NiFeO_x_ retains about 44% of its initial photocurrent, whereas the unmodified BiVO_4_ maintains approximately 50%. Although the co-modified electrode demonstrates substantially improved initial performance, its long-term stability is slightly lower than that of the unmodified sample. This decline can be primarily attributed to chemical corrosion and interfacial degradation under the alkaline electrolyte environment (pH ~ 9.5). The NiFeO_x_ cocatalyst offers partial protection by facilitating hole extraction and reducing surface recombination; however, it may not provide sufficient long-term passivation against electrochemical dissolution or catalyst delamination. These findings suggest that while surface modification enhances PEC activity, ensuring robust structural and chemical stability under operating conditions remains a critical challenge.

## 4. Conclusions

In this study, a cost-effective and simplified method is developed for the fabrication of high-performance photoanodes, which holds great promise for practical PEC applications. Monoclinic-phase BiVO_4_ photoanodes are successfully synthesized via a simplified SILAR technique, followed by boron doping and PEC deposition of NiFeO_x_ to construct B/BiVO_4_/NiFeO_x_ heterostructured photoanodes with enhanced surface properties. Comprehensive material characterizations, including SEM, XRD, and XPS, confirm the successful synthesis of monoclinic BiVO_4_ and the uniform distribution of B, Ni, and Fe elements on the photoanode surface, indicating effective doping and surface modification. Among the fabricated photoanodes, B/BiVO_4_/NiFeO_x_ exhibit the highest PEC activity, achieving a photocurrent density of 2.45 mA cm^−2^ at 1.23 V vs. RHE, which is 5.3 and 3.6 times higher than that of pristine BiVO_4_ and B/BiVO_4_, respectively. Furthermore, this heterostructure delivers a high ABPE of 0.77% at 0.66 V vs. RHE, with a charge separation efficiency of up to 79.5% at 1.23 V vs. RHE. This work provides new insights into the rational design of cost-efficient and high-performance photoanodes, offering promising potential for scalable solar-driven hydrogen production.

## Figures and Tables

**Table 1:** Main experimental Instruments.

Instrument	Model	Manufacturer
Precision Electronic Balance	SQP	Sartorius Scientific Instruments Co., Ltd., Beijing, China
High-Resolution X-ray Diffractometer	Panalytical	Malvern Panalytical, Almelo, The Netherlands
Scanning Electron Microscope	TESCAN MIRA LMS	TESCAN (China) Co., Ltd., Brno, Czech Republic/Shanghai, China
X-ray Diffractometer	MiniFlex-600	Rigaku Corporation, Tokyo, Japan
UV-Visible Spectrophotometer	UV-3600 PLUS	Shimadzu Corporation, Kyoto, Japan
X-ray Photoelectron Spectrometer	Thermo Scientific K-Alpha	Thermo Fisher Scientific, Waltham, MA, USA
Xenon Lamp	PLS-SXE300+	Beijing Pofilai Technology Co., Ltd., Beijing, China
Muffle Furnace	KSL-1100X	Hefei Kejing Material Technology Co., Ltd., Hefei, China
Electrochemical Workstation	CHI 760E	Shanghai Chenhua Instruments Co., Ltd., Shanghai, China
Digital Constant Temperature Magnetic Stirrer	85-2	Changzhou Yuexin Instrument Manufacturing Co., Ltd., Changzhou, China
pH Tester	PH-10	Lichen Instruments, Shanghai, China

**Table 2:** Elemental content of B/BiVO_4_/NiFeO_x_ materials.

Elements	Weight Percentage (%)	Atomic Percentage (%)
B	24.12	57.6
O	16.04	25.89
Bi	34.63	4.28
V	15.13	7.67
Fe	5.83	2.70
Ni	4.24	1.87
Total	99.99	99.99

**Table 3:** EIS fitting data for each photoelectrode.

Photoelectrode	R1 (Ω)	Rct (Ω)
BiVO_4_	111.3	3370
B/BiVO_4_	70.69	503.4
B/BiVO_4_/NiFeO_x_	66.25	343.8

## Data Availability

All the datasets used in this manuscript are publicly available datasets already in the public domain.
